# Variability of 18rDNA loci in four lace bug species (Hemiptera, Tingidae) with the same chromosome number

**DOI:** 10.3897/CompCytogen.v9i4.5376

**Published:** 2015-09-02

**Authors:** Natalia V. Golub, Viktor B. Golub, Valentina G. Kuznetsova

**Affiliations:** 1 Zoological Institute, Russian Academy of Sciences, Universitetskaya nab. 1, St. Petersburg 199034, Russia; 2 Voronezh State University, Universitetskaya pl. 1, Voronezh, 394006, Russia

**Keywords:** Karyotype, sex chromosomes, FISH, rDNA, (TTAGG)*_n_*, Hemiptera, Heteroptera, Cimicomorpha, Tingidae

## Abstract

Male karyotypes of *Elasmotropis testacea* (Herrich-Schaeffer, 1835), *Tingis cardui* (Linnaeus, 1758), *Tingis crispata* (Herrich-Schaeffer, 1838), and *Agramma femorale* Thomson, 1871 (Heteroptera, Cimicomorpha, Tingidae) were analyzed using conventional chromosome staining and FISH with 18S rDNA and (TTAGG)*_n_* telomeric probes. The FISH technique was applied for the first time in the Tingidae. In spite of the fact that all species showed the same chromosome number (2n = 12 + XY), they have significant differences in the number and position of rDNA loci. FISH with the classical insect (TTAGG)*_n_* probe produced no signals on chromosomes suggesting telomeres in lace bugs to be of some other molecular composition. Tingidae share absence of the (TTAGG)*_n_* telomeric sequence with all so far studied taxa of the advanced true bug infraorders Cimicomorpha and Pentatomomorpha.

## Introduction

Tingidae (lace bugs) are a large widespread family of herbivorous bugs including 2200 species belonging to 280 genera. The family is subdivided into two, Tinginae and Cantacaderinae, or into three (Vianaidinae in addition) recent subfamilies; the subfamily Tinginae is the largest and the most diverse subfamily of lace bugs. Tingidae are placed in the Cimicomorpha, but their relationships within this large infraorder are not entirely clear (Golub and Popov 2012, Golub et al. 2012).

Many studies have proven that chromosome alterations are significant for species evolution and then, cytogenetics can be a useful tool for evolutionary, taxonomic, phylogenetic and speciation studies (White 1973, King 1993).

Cytogenetic data on members of the Tingidae are scarce and only involve species of the Tinginae. Currently, chromosome information of 29 species, belonging to 18 genera, i.e., approximately 1% and 6% respectively is known (Ueshima 1979, Nokkala and Nokkala 1984, Grozeva and Nokkala 2001). With one exception (see Discussion), the karyotypes of the species studied are similar in that they include six pairs of autosomes.

All previous investigations of lace bugs have been carried out using conventional chromosome staining techniques. Identification of individual chromosomes in karyotypes is a difficult task in the case of true bugs because of morphologically uniform holokinetic chromosomes. However, with the use of C-banding technique, Grozeva and Nokkala (2001) were successful in identifying separate chromosomes in 13 lace bugs species and revealing differences between them in C-band pattern. These findings showed that C-heterochromatin distribution has had a major role in the karyotype evolution of the family Tingidae.

In the past decades, fluorescence in situ hybridization (FISH) has increased the resolution of the true bugs’ cytogenetics. Thanks to this technique, the analysis of the karyotypes has become more informative and comprehensive. In true bugs, ribosomal genes are commonly used as markers for the physical mapping of their chromosomes (reviewed in Grozeva et al. 2014).

Here, the first FISH-based study for the characterization of tingid karyotypes is presented. We describe the karyotypes of *Elasmotropis testacea* (Herrich-Schaeffer, 1835), *Tingis cardui* (Linnaeus, 1758), *Tingis crispata* (Herrich-Schaeffer, 1838), and *Agramma femorale* Thomson, 1871 after FISH with an 18S rDNA probe. Note that for two last species, the standard karyotype is reported for the first time.

Additionally, we used FISH with a (TTAGG)*_n_* probe to analyze whether the classical “insect” telomeric motif (TTAGG)*_n_* is present in the lace bug species. Previous studies on species of two cimicomorphan families (Miridae and Cimicidae) showed the absence of this telomeric repeat (Frydrychová et al. 2004, Grozeva et al. 2011).

## Material and methods

The material studied is presented in Table 1.

**Table 1.** Material used for chromosome analysis.

Species	Number of males/chromosome plates studied	Locality and date of collection	Host plant
*Elasmotropis testacea*	2/37	Russia, Republic of Bashkortostan, South-Ural state natural reserve, env. of village Terekly, 12 km ENE of settl. Arhangelskoe, 54°26'N, 56°57'E, alt. 269 m, 5.08.2014	*Echinops* sp. (Asteraceae)
*Tingis cardui*	2/19	Russia, Republic of Bashkortostan, South-Ural state natural reserve, env. of settl. Inzer, 54°13'N, 57°34'E, alt. 349 m, 4.08.2014	*Carduus* sp. (Asteraceae)
*Tingis crispata*	3/143	Russia, Tolyatti, 53°31'N, 49°25'E, alt. 95 m, 13.08.2014	*Artemisia vulgaris* Linnaeus, 1753 (Asteraceae)
*Agramma femorale*	2/23	Russia, Republic of Bashkortostan, South-Ural state natural reserve, env. of village Revet’, 54°11'N, 57°37'E, alt. 285 m, 10.08.2014	*Juncus* sp. (Juncaceae)

Lace bug species were collected in 2014 by V. Golub in Republic of Bashkortostan, Russia. Only male adult specimens were analyzed. In field, the specimens were fixed immediately after capturing in 3:1 fixative (96% ethanol: glacial acetic acid) and stored at 4 °C. In laboratory, testes were dissected in a drop of 45% acetic acid and squashed. The cover slips were removed using dry ice. Prior to staining, the preparations were examined by phase contrast microscopy. Chromosome staining techniques applied were a Feulgen-Giemsa method as described in Grozeva and Nokkala (1996) and fluorescence *in situ* hybridization (FISH) with 18S rDNA and (TTAGG)*_n_* telomeric probes. 18S rDNA and (TTAGG)*_n_* probe preparation and hybridization were carried out as described in Grozeva et al. (2010, 2014). In brief, chromosome preparations were treated with 100 µg/ml RNaseA and 5 mg/ml Pepsin solution to remove excess RNA and proteins. Chromosomes were denatured on a slide in hybridization mixture with biotinylated 18S rDNA probe from the genomic DNA of *Pyrrhocoris apterus* (Linneus, 1758) and rhodaminated (TTAGG)*_n_* probe with addition of salmon sperm DNA blockage and then hybridized for 36 h. Hybridization signals were detected with NeutrAvidin-FITC. Chromosomes were mounted in an antifade medium (ProLong Gold antifade reagent with DAPI, Invitrogen) and covered with a glass coverslip. Chromosome slides were analyzed under a Leica DM 6000 B microscope; images were taken with a Leica DFC 345 FX camera using Leica Application Suite 3.7 software with an Image Overlay module.

## Results

### Conventional staining and FISH with an 18S rDNA probe

*Tingis crispata*, 2n = 14 (12A + XY)

Published data: absent

**Figures 1–10.**
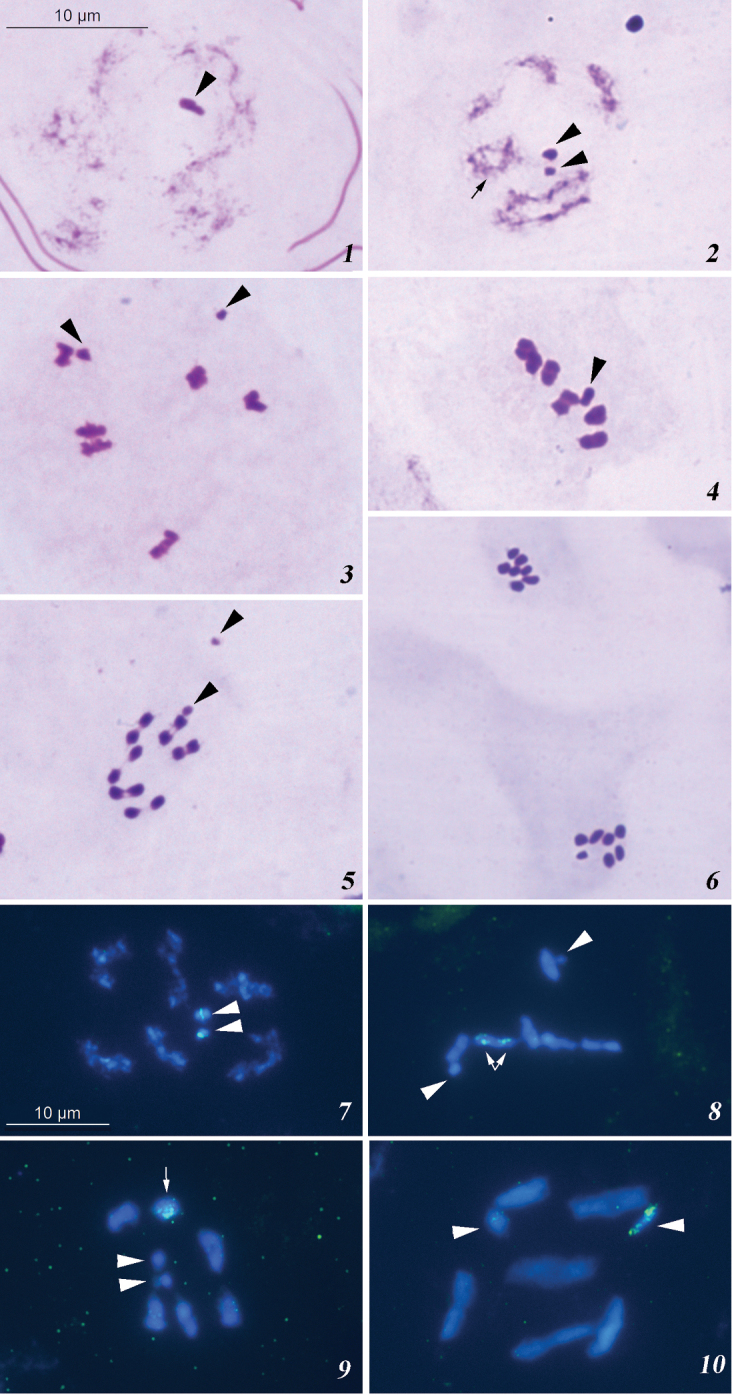
**1–6** Male meiosis in *Tingis crispata* (conventional staining): **1** diffuse stage **2** early diakinesis, two-chiasmate bivalent is indicated by arrow **3** early MI **4** mature MI **5** early AI **6** MII. Sex chromosomes are indicated by arrowheads **7–10** Meiotic chromosomes in Tingidae species after FISH with an 18S rDNA probe: **7** diakinesis in *Tingis crispata* **8** first prometaphase in *Tingis cardui* **9** MI in *Elasmotropis testacea* **10** first prometaphase in *Agramma femorale*. Sex chromosomes are indicated by arrowheads; autosomally located signals are indicated by arrows.

During the diffuse stage, the autosomes were de-condensed whilst the X and Y chromosomes appeared to be fused and heteropycnotic (Fig. 1). Early diplotene (Fig. 2) revealed six autosomal bivalents, each with one, rarely two chiasmata, and the X and Y chromosomes positioned close to each other. The bivalents gradually decreased in size, and sex chromosomes were of different size. At early metaphase I (MI), sex chromosomes were seen well apart from each other (Fig. 3) whilst at mature MI they formed a heteromorphic pseudobivalent (Fig. 4). At early anaphase I, sex chromosomes segregated ahead of the autosomal bivalents (Fig. 5). At MII, the two daughter nuclei, each with seven elements, namely, 6 autosomes and either the X or the Y chromosome, were present (Fig. 6).

The 18S rDNA FISH resulted in appearance of a comparatively small interstitial signal in the larger sex chromosome (presumably, the X) and a larger subterminal signal in the smaller sex chromosome (presumably the Y) (Fig. 7).

*Tingis cardui*, 2n = 14(12A + XY)

Published data: 2n = 14(12A + XY) (Southwood and Leston 1959)

At first prometaphase subjected to 18S rDNA FISH, eight elements were present, including six autosomal bivalents and X and Y chromosomes which lied separately from each other. The bivalents constituted a series decreasing in size, and sex chromosomes were of different size. The subterminally located 18S rDNA sites were revealed on both homologues of a medium-sized autosomal bivalent (Fig. 8).

*Elasmotropis testacea*, 2n = 14(12A + XY)

Published data: 2n = 14(12A + XY) (Grozeva and Nokkala 2001)

At first metaphase subjected to 18S rDNA FISH, eight elements were present, including six autosomal bivalents which formed a ring with a pseudobivalent of the X and Y chromosomes located in its center. The bivalents constituted a series decreasing in size, and sex chromosomes were of similar size. The subterminally located 18S rDNA sites were revealed in a medium-sized bivalent (Fig. 9).

*Agramma femorale*, 2n = 14(12A + XY)

Published data: absent

At first prometaphase subjected to 18S rDNA FISH, eight elements were present, including six autosomal bivalents and X and Y chromosomes which lied separately from each other. The bivalents constituted a series decreasing in size, sex chromosomes could not be told apart because of their similar size. The 18S rDNA signals were dispersed all over one of the two sex chromosomes (Fig. 10).

#### FISH with a (TTAGG)_n_ telomeric probe

In none of the species studied, the (TTAGG)*_n_* telomeric probe produced fluorescent signals.

## Discussion

Like other true bugs, Tingidae have holokinetic chromosomes (Ueshima 1979, Kuznetsova et al. 2011). These chromosomes possess diffuse or non-localized centromeres and can therefore display a unique capability for karyotype evolution via occasional fusion/fission events (White 1973). In spite of this, both previous cytogenetic investigations (Ueshima 1979, Nokkala and Nokkala 1984, Grozeva and Nokkala 2001) and our new data suggest that Tingidae are characterized by a stable number of autosomes, 12 in diploid complements. The only exception seems to be *Acalypta parvula* (Fallén, 1807) which has, according to Southwood and Leston (1959), 2n = 12(10A + XY) in a population from British Isles. However males of this species from Finland were reported to have 2n = 12A + X (Grozeva and Nokkala 2001). Assuming these chromosome data are correct, one can suggest the existence of two species hidden under one species name. The majority of hitherto studied lace bug species, namely 25 of the 29, possess a XY/XX type of sex determination. This sex chromosome system was suggested to represent a plesiomorphic state in the Heteroptera, and the sporadic occurrence of X(0) bed bug species to be due to repeated loss of the Y chromosome, i.e. a result of convergent evolution (homoplasy) (Nokkala and Nokkala 1983, 1984, Kuznetsova et al. 2011, Grozeva et al. 2014). Such a loss has also occurred at least twice within the Tingidae: in the genera *Acalypta* Westwood, 1840 and *Kalama* Puton, 1876. All the three studied *Acalypta* species, namely, *Acalypta carinata* (Panzer, 1806), *Acalypta nigrina* (Fallén, 1807), and most likely also *Acalypta parvula* (Grozeva & Nokkala, 2001), and a single studied *Kalama* species, namely *Kalama tricornis* Schrank, 1801 (Nokkala and Nokkala 1984: as *Dictyonota tricornis* (Schrank, 1801), Grozeva and Nokkala 2001), were found to have a derived system X(0).

For insects with holokinetic chromosomes the low number of chiasmata is characteristic and is considered as a result of a specific structure of holokinetic bivalents (Nokkala et al. 2004). In Tingidae, one or occasionally two chiasmata in every bivalent were described (Ueshima 1979, Grozeva and Nokkala 2001). This pattern is also revealed in the four species here examined. Within Cimicomorpha, Tingidae share male chiasmate meiosis with Reduviidae (Ueshima 1979), whereas other families for which such evidence is available, namely, Microphysidae, Nabidae s.str., Anthocoridae s.str., Cimicidae, and Miridae, seem to have achiasmate meiosis in males (Kuznetsova et al. 2011).

In “standard” meiosis, during the first division all the chromosomes reduce in number (reductional division), whereas during the second division the chromatids separate (equational division), and this pattern is named “pre-reduction” (White 1973). However Heteroptera show an inverted sequence of meiotic divisions for sex chromosomes in males, the so-called “sex chromosome post-reduction”. It means that, unlike autosomes, the sex chromosome(s) divide equationally at anaphase I and reductionally at anaphase II. On very rare occasion, in individual bug species, a pre-reductional division of sex chromosomes was observed, and such species have also been reported within cimicomorphan families Miridae (Grozeva et al. 2006, 2007) and Reduviidae (Manna and Deb-Mallick 1981). Importantly, lace bugs are the only heteropteran family showing pre-reduction of sex chromosomes in spermatogenesis of all the studied species (Ueshima 1979, Grozeva and Nokkala 2003, present study). Since all other members of the Hemiptera invariably display pre-reduction, the sex chromosomes’ post-reduction can be considered as an autapomorphy of true bugs without Tingidae.

In groups with holokinetic chromosomes, the main problem is to identify individual chromosomes and chromosomal regions in karyotypes. Different cytogenetic techniques, e.g. C-banding, DNA-specific fluorochrome staining, AgNO_3_ staining, make possible only a few markers to be revealed in true bugs’ karyotypes (Papeschi and Bressa 2006, Kuznetsova et al. 2011). Regarding the Tingidae, a single work aimed to reveal differences between species in C-banding pattern was published by Grozeva and Nokkala (2001). The 13 studied species belonging to 10 genera were found to differ in the number (from one to eight per haploid complement) and location (terminal, interstitial or both) of bands on both autosomes and sex chromosomes. The data obtained showed that a quite substantial redistribution of chromosome material within chromosomes occurred during the evolution of this group without chromosome fragmentation or fusions (Grozeva and Nokkala 2001). Thus, the species-specific organization of the constitutive heterochromatin can be used as an additional cytogenetic marker for the lace bug species differentiation.

In order to reveal additional chromosomal markers and gain deeper insights into the evolution of the Tingidae, we have applied FISH with 18S rDNA and telomeric (TTAGG)*_n_* probes to the four species from the present study. This is the first time that the lace bugs have been the subject of a molecular cytogenetic study. Physical location of genes remains very poorly studied in true bugs. Out of approximately 40.000 described species (Weirauch and Schuh 2011), only 94 species have been investigated in this respect and only the rRNA genes and telomeric sequences were mapped (Grozeva et al. 2014). The species studied belong to 38 genera, 10 families, and three (out of 8) infraorders including Nepomorpha (Belostomatidae), Pentatomomorpha (Coreidae, Lygaeidae, Pentatomidae, and Pyrrhocoridae), and Cimicomorpha (Cimicidae, Largidae, Miridae, Reduviidae, and Rhopalidae). The sites for rRNA at a rate of one to four (per diploid genome) were found to locate variously in different species: either on autosomes (the largest or one of the medium-sized pairs), or on m-chromosomes, or on sex chromosomes (X or both X and Y) or on both a pair of autosomes and the X-chromosome. The autosomal location seems to predominate being found in half of the species studied. The majority of rDNA sites show a terminal localization, however in rare cases they are positioned interstitially in chromosomes. The most impressive variation regarding the number and the type of chromosomes (autosomes and/or sex chromosomes) that carried the rRNA genes is described in the kissing bug subfamily Triatominae (Cimicomorpha: Reduviidae) even though it demonstrates a highly conserved karyotype including 20 autosomes in the great majority of studied species (Panzera et al. 2012, 2014, Pita et al. 2013).

A very similar variation holds for the four tingid species possessing the same karyotype, 2n = 12 + XY, including two closely related species of the genus *Tingis* Fabricius, 1803. Our findings suggest that chromosomal divergence can occur among seemingly conserved karyotypes and may play a role in reproductive isolation and speciation of the family Tingidae. Males of *Tingis crispata* were found to have rDNA sites on both sex chromosomes, interstitial on the larger and subterminal on the smaller. Since in the XY true bugs species the larger of the two sex chromosomes is conventionally taken as the X (e.g. Ueshima 1979, Grozeva et al. 2014), we suggested that this is also the case in *Tingis crispata*. In contrast, males of *Tingis cardui* showed subterminally located sites on one medium-sized pair of autosomes. In the two remaining species, *Elasmotropis testacea* and *Agramma femorale*, ribosomal genes were found on a medium-sized autosomal pair (located subterminally) and on one of the two homomorphic sex chromosomes (multiple sites), respectively.

Changes in the number and location of rDNA loci are a well-known phenomenon in eukaryotic organisms, including true bugs (e.g. Panzera et al. 2012, Grozeva et al. 2014). As regards the ability of rDNA clusters to move and vary in number among the closely related species with the same chromosome number, different mechanisms have been suggested, including structural chromosome rearrangements (inversions and translocations), transposition, ectopic recombination, transposable elements (Panzera et al. 2012, Pita et al. 2013, Grozeva et al. 2014) and even a homoploid hybrid speciation, i.e. hybridization without a change in chromosome number (referenced in Vershinina et al. 2015). In Triatominae bugs, the occurrence of heterologous associations among non-homologous autosomes and heterochromosomes seems to favor the transposition and ectopic recombination hypotheses (Panzera et al. 2012). However, much more work is needed to identify mechanisms responsible for the ribosomal loci variation in lace bugs.

The majority of insect species is known to share the telomeres composed of the pentanucleotide TTAGG repeat which is considered as an ancestral telomeric motif in this large group of Arthropoda (Frydrychová et al. 2004, Vitková et al. 2006). Many higher level insect groups preserved this telomeric sequence, but some of them have lost it during the evolution. Recently, it has been shown that in Heteroptera, the classical insect (TTAGG)*_n_* telomeric sequence is absent in the evolutionarily advanced families Miridae, Cimicidae (Cimicomorpha), Pyrrhocoridae and Pentatomidae (Pentatomomorpha) (Frydrychová et al. 2004. Grozeva et al. 2011) but is present in the family Belostomatidae from a more basal infraorder Nepomorpha (Kuznetsova et al. 2012). According to our data, this telomeric sequence is absent in all the four examined lace bug species and probably in the family Tingidae as a whole. This new finding reinforces the hypothesis that the (TTAGG)*_n_* telomeric motif was lost during the evolution of the Heteroptera, at least in the common ancestor of large infraorders Pentatomomorpha and Cimicomorpha (Kuznetsova et al. 2012).
